# Monetary Reward and Punishment to Response Inhibition Modulate Activation and Synchronization Within the Inhibitory Brain Network

**DOI:** 10.3389/fnhum.2018.00027

**Published:** 2018-03-01

**Authors:** Rupesh K. Chikara, Erik C. Chang, Yi-Chen Lu, Dar-Shong Lin, Chin-Teng Lin, Li-Wei Ko

**Affiliations:** ^1^Department of Biological Science and Technology, National Chiao Tung University, Hsinchu, Taiwan; ^2^Brain Research Center, National Chiao Tung University, Hsinchu, Taiwan; ^3^Institute of Cognitive Neuroscience, National Central University, Taoyuan, Taiwan; ^4^Institute of Bioinformatics and Systems Biology, National Chiao Tung University, Hsinchu, Taiwan; ^5^Department of Pediatrics, Mackay Memorial Hospital, Taipei, Taiwan; ^6^Faculty of Engineering and Information Technology, University of Technology Sydney, Sydney, NSW, Australia

**Keywords:** response inhibition, no-feedback, reward, punishment, motivation, posterior cingulate gyrus, functional magnetic resonance imaging, electroencephalography

## Abstract

A reward or punishment can modulate motivation and emotions, which in turn affect cognitive processing. The present simultaneous functional magnetic resonance imaging-electroencephalography study examines neural mechanisms of response inhibition under the influence of a monetary reward or punishment by implementing a modified stop-signal task in a virtual battlefield scenario. The participants were instructed to play as snipers who open fire at a terrorist target but withhold shooting in the presence of a hostage. The participants performed the task under three different feedback conditions in counterbalanced order: a reward condition where each successfully withheld response added a bonus (i.e., positive feedback) to the startup credit, a punishment condition where each failure in stopping deduced a penalty (i.e., negative feedback), and a no-feedback condition where response outcome had no consequences and served as a control setting. Behaviorally both reward and punishment conditions led to significantly down-regulated inhibitory function in terms of the critical stop-signal delay. As for the neuroimaging results, increased activities were found for the no-feedback condition in regions previously reported to be associated with response inhibition, including the right inferior frontal gyrus and the pre-supplementary motor area. Moreover, higher activation of the lingual gyrus, posterior cingulate gyrus (PCG) and inferior parietal lobule were found in the reward condition, while stronger activation of the precuneus gyrus was found in the punishment condition. The positive feedback was also associated with stronger changes of delta, theta, and alpha synchronization in the PCG than were the negative or no-feedback conditions. These findings depicted the intertwining relationship between response inhibition and motivation networks.

## Introduction

Motivation plays an essential role in human behaviors. A significant amount of studies have revealed that the reward prospect has noticeable effects on a range of cognitive functions such as working memory (Gilbert and Fiez, 2004; Beck et al., 2010), memory development (Adcock et al., 2006), and visual attention (Krebs et al., 2009; Padmala and Pessoa, 2011; Stoppel et al., 2011; Schevernels et al., 2014). Many extant studies attempted to modulate motivation by using a cue representing a reward conditional on the correct response in the upcoming task (Knutson et al., 2000; Rosell-Negre et al., 2014). Cognitive functions such as action–effect binding (Krebs et al., 2009) and conflict adaptation (Braem et al., 2012) can be changed for separate reward trials in a retarded situation. These outcomes suggest that a prospect of reward can result in an overall state of constantly planned, proactive control (Locke and Braver, 2008), namely enhanced top-down regulatory mechanisms (Pessoa and Engelmann, 2010; Chelazzi et al., 2013).

Among the functions subject to the impact of motivation, response inhibition stands out as a particularly interesting case (Scheres et al., 2001; Rosell-Negre et al., 2014). Response inhibition refers to the suppression of a to-be-executed action and is generally considered to be a part of cognitive control, which is essential for adaptive human behaviors. It is commonly evaluated with the stop-signal task, in which participants are instructed to respond to a Go signal by default, but to withhold the response upon the presence of an infrequent Stop signal following the Go signal (see Logan et al., 2014 for an updated review). A dominant and successful model explaining the potential process underlying stop-signal task is the horse-race model, which asserts that the performance in a stop trial is essentially the outcome of the competition between the independent processing of go and stop signals (Boucher et al., 2007; Logan et al., 2014). The competition between the signals can be tuned through adjusting the temporal delay between the go and stop stimuli. While a successfully inhibited response has no observable reaction time, a variety of methods considering both the stop signal delay (SSD) and reaction time can be applied to estimate the temporal latency of response inhibition, namely the stop signal response time (SSRT).

In the few studies exploring how motivation modulates response inhibition, it has been demonstrated that rewarding the go or the stop response has distinct consequences on the performance in the stop-signal task. On the one hand, there are studies showing that response inhibition is compromised when reward contingencies encourage the go response (Padmala and Pessoa, 2010). Increasing the probability to receive a reward led to decrease in SSRT, indicating enhanced proactive inhibitory control, and brain activation was stronger in the frontal, parietal, and subcortical areas under reward-stimulated response inhibition (Scheres et al., 2001; Rosell-Negre et al., 2014). On the other hand, reduced SSRT has been found when the reward is associated with stop trials, suggesting that prospect of reward can enhance successful response inhibition (Boehler et al., 2012, 2014), and increase activation in the inferior frontal gyrus (IFG), right insula, dorsal anterior cingulate cortex, and pre-supplementary motor area (pre-SMA) in reward associated stop trials (Boehler et al., 2014).

Studies investigating the neural mechanisms of reward and punishment could shed light on how motivation may influence response inhibition in general. For example, Enzi et al. (2015) asked both healthy participants and long-term cannabis users to perform a monetary incentive delay task, a reliable experimental model that enables a differentiation between the reward and punishment processing. They found that the brain regions typically involved in the processing of reward and punishment in cannabis users include the bilateral IFG, bilateral striatum, and bilateral anterior insula as compared to healthy participants. Drueke et al. (2015) examined the scenario when both negative and positive effects can appear following choice responses, and reported higher striatal and amygdala activation in positive than in negative feedback, while no brain regions were more strongly activated for the opposite. It also has been shown that the feedback during monetary reward (i.e., positive feedback) and punishment (i.e., negative feedback) can affect the emotion and motivation and stimulates the corresponding brain regions such as insular cortex, orbitofrontal, medial prefrontal cortex, striatal, and amygdala (Tsukamoto et al., 2006; Drueke et al., 2015).

While previous functional magnetic resonance imaging (fMRI) studies have attributed reward enhanced behavioral performance to improved reactive control process, as shown through reward associated activations in the prefrontal and right-lateral central areas (Boehler et al., 2014), little information has been reported regarding the role of emotion and motivation in response inhibition. Moreover, very few studies have explored how motivational factor modulate performance of the stop signal task using reward contingencies (cf. Leotti and Wager, 2010; Padmala and Pessoa, 2010; Boehler et al., 2012, 2014). The extant behavioral studies of response inhibition have found that participants showed longer SSRT in the reward than the no-reward condition, suggesting increased difficulty in inhibiting responses in reward condition (Padmala and Pessoa, 2010). Additionally, reduced activation has been found for successful inhibition of response during reward condition in frontal regions of the brain such as left precentral cortex and bilateral IFG, in dorsal striatal areas including bilateral putamen, and in parietal cortex, such as the bilateral intraparietal sulcus and the inferior parietal gyrus (Padmala and Pessoa, 2010). Nevertheless, no study has investigated brain activities pertaining to reward and punishment in the context of stop-signal paradigm within the same group of participants. Therefore, the current study takes on this approach and aims to render a sounder picture of how positive and negative motivational factors modulate proactive inhibitory control processes in the brain.

Though a previous fMRI study has demonstrated enhanced activation in medial prefrontal cortex associated with reactive control in reward-related stop trials (Boehler et al., 2014), with respect to the timing of different processing steps in reward and punishment related stop signal task, fMRI may not be sensitive enough to detect the brief fluctuations in attentional processes. To this end, we take advantage of simultaneously recording electroencephalography (EEG) signals in the current fMRI experiment. Previous studies have utilized ERP analysis in the stop signal task, which have focused on the frontal N2 and P3 waves, defined the N2/P3 complex, which is related to response inhibition (Eimer, 1993; Bokura et al., 2001; Van Boxtel et al., 2001; Kok et al., 2004; Bekker et al., 2005; Ramautar et al., 2006; Schmajuk et al., 2006; Huster et al., 2010, 2011; Van Gaal et al., 2011). A former study found that the increase N1 and P3 waves in the frontal cortex with reward under response inhibition (Schevernels et al., 2015). However, there are also a few studies adopting frequency-power analyses to demonstrate a standard function of attentional processes in the stop signal task (Boehler et al., 2009; Schevernels et al., 2015). To further precisely identify the time-based neural signatures for inhibitory control, an another method of investigating EEG signal is using time and frequency measure for recognition the brain oscillations components involved in response inhibition (Herrmann et al., 2005). Moreover, Basar et al. (1999) established that the EEG signals could be examined in the frequency domain and fluctuations of precise frequencies are associated with particular cognitive functions, such as alpha (8–12 Hz) band oscillations in both continued and focused attention (Mathewson et al., 2014).

In the current study, we adopted simultaneous fMRI and EEG recording with high-temporal and high-spatial resolutions, respectively, to identify modulations in the human brain during response inhibition under contexts of either reward or punishment settings. Matching independent recordings of EEG and fMRI signals, simultaneous fMRI-EEG studies characterize functional stimulations and frequency fluctuations of brain dynamics during similar experimental circumstances and are thus more likely to capture signals from similar brain activities (Huster et al., 2012; Mulert, 2013). This study aims to investigate the neural mechanisms of response inhibition during monetary reward (i.e., for correct stop trials) and punishment (i.e., for fail stop trials) with simultaneous recording of fMRI and EEG. To accomplish this aim, we adopted a modified stop signal task. In the current experiment, scenes from a famous shooting game (Counter-Strike) were adopted as the graphic backdrop to form a battlefield scenario (BFS), where a picture of a terrorist holding a weapon was used as the go stimulus, and a hostage picture used as the stop stimulus. We predict the following findings: (1) the adaptive monetary reward will enhance the participants’ performance under response inhibition; (2) reward contingencies should enhance the activation of the brain regions involved in response inhibition processes including the left IFG (lIFG) and pre-SMA; and finally (3) positive performance feedback would elicit higher brain activation compared to negative performance feedback.

## Materials and Methods

### Participants

Twenty participants (18 males and 2 females; mean ± SD age: 23.3 ± 2.4 years) took part in this study. All the participants were right-handed, had normal or corrected-to-normal vision and no history of psychiatric or neurological disorders. Before the experiment, each participant provided a written informed consent. After finishing the experimental task, each participant received a compensation of 800 NTD (New Taiwan Dollar) and an additional performance-dependent bonus of 10 NTD per trial. The experimental protocol was approved by the Research Ethics Committee of the National Taiwan University, Taipei, Taiwan.

### Experimental Design

**FIGURE 1:**
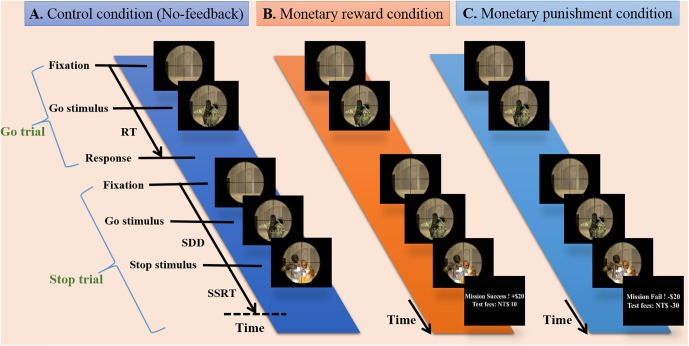
Experimental design. Presentation of stimuli during three conditions including no-feedback, reward, and punishment on the battlefield scene. **(A)** The no-feedback condition was used as a control condition. **(B)** In the reward condition, minimum compensation was started from 800 NTD, and each successful withheld response aggregates an additional bonus of 10 NTD (i.e., positive feedback). **(C)** In the punishment condition, each failure in withholding response aggregates a penalty of 10 NTD (i.e., negative feedback).

The participants were instructed to respond to the go stimulus and withhold their responses in the presence of the stop signal, which appeared as a hostage image at some temporal delay after the go stimulus. All participants completed three runs of simultaneous fMRI-EEG recording, and each run was evenly divided into blocks of no-feedback, reward and punishment conditions (see **Figure 1**), where the order of conditions was Latin-square counterbalanced across runs. The no-feedback condition was used as a control condition according to a previous study (Ko et al., 2016). At the beginning of the experiment, each participant was told to have a credit of 800 NTD. As the experiment proceed, each successful withheld response was encouraged with an additional bonus of 10 NTD in the reward condition, and each failure in withholding the response resulted in a penalty of 10 NTD in the punishment condition. Note that participants only receive positive feedback in the reward block and only negative feedback in the punishment block.

Each run had a total of 210 trials. Twenty-five percent of the trials (within each condition block) were stop-trials and the rest were go-trials. Each go-trial started with a fixation cross lasting for a random period of time (0.5–6.5 s), followed by a signal for 1 s or up to feedback. In a stop-trial, the stop signal was presented at the assigned SSD after the go-signal. The critical SSD (cSSD), which resulted in a 50% chance of successful stop (SS), was determined using a staircase procedure before participants entered the MR scanner. The staircase tracking system operated as follows: the SSD started at 150 ms, and each SS trial led to an increase in the SSD for 50 ms; on the contrary, the SSD was reduced by 50 ms for each failed stop (FS). The lower bound of the SSD was 150 ms. The staircase tracking procedure terminated after 52 reversals, and the cSSD is the average of the SSDs at all of the reversal points. We utilized five different SSDs (i.e., cSSD, cSSD ± 40 ms, and cSSD ± 80 ms), and each SSD had an equal number of trials.

### Acquisition and Preprocessing of fMRI Data

All participants completed the experiment in a Siemens 3T MAGNETOM Skyra scanner situated in the Mind and Brain Imaging Center at the National Chengchi University, Taipei, Taiwan. Functional brain images were obtained through a gradient echo-planar imaging sequence (TR: 2000 ms; TE: 25 ms; flip angle: 90°; matrix size: 64 × 64; field of view: 220 mm × 220 mm; voxel size: 3.438 mm × 3.438 mm × 4.0 mm; 292 volumes per run). Structural T1-weighted pictures were obtained using the MP-RAGE sequence (TR: 2530 ms; TE: 3.03 ms; flip angle: 7°; matrix size: 224 × 256; field of view: 224 mm × 256 mm; slice thickness: 1 mm; 192 slices; in-plane resolution: 1 mm × 1 mm). The flow of all preprocesses and the statistical analysis were conducted with the analysis of functional neuroimages (AFNI software, version 2016-11-03; Cox, 1996). The preprocessing stream involved slice time correction (time-shifting the time series using Fourier interpolation), image reconstruction and motion correction (linear least-squared alignment by affine transformation with three translational parameters and three rotational parameters). Edge recognition methods eliminated stimulation or activation outside the brain. At the end of the preprocessing, the anatomical image of each participant was transformed into a standard space (the MNI 152 brain template) by an automated feature adaptation algorithm (Collins et al., 1994). The functional data of each participant were initially aligned to their anatomical images and then converted into the normalized MNI space.

### Acquisition and Preprocessing of EEG Signal

A 34-channel amplifier compatible with MR (Brain Products, Brainamp MR) and an MR compatible EEG cap with 32 standard channels were used. EEG signals were collected in the MR scanner simultaneously with the acquisition of fMRI. The EEG cap had 31 channels for recording the brain activities and one for electrocardiogram (ECG) recording. The skin impedance of the channel was saved less than 10 kOhm by the use of abrasive electrolytic gel (ABRALYT HiCl). Fiber optic cables transferred the EEG signals to an IBM-compatible laptop computer and acquired through the BrainVision (BrainVision Recorder, Brain Products) program synchronized with the BOLD signals by MR scanner triggers. EEG signals were acquired through a 1–250 Hz passband and were digitized at 5000 Hz with 32-bit resolution (equal to 0.5 μV, dynamic range: 16.38 mV). After that, the MR tilt artifacts were removed in the EEG signal. The MRI-denoised EEG signals were down sampled at 500 Hz, and ECG recording cardio balance signals were used to correct the EEG signals by wave-recognition algorithms in the brain-vision analyzer software. These algorithms used a wide dynamic range to capture both low amplitude EEG and large MRI artifacts without distortion, 5 kHz sampling and low pass filtering before to the main gain stage. The MRI artifact was reduced by subtracting an averaged artifact waveform, followed by adaptive noise cancelation to reduce any residual artifact. All these procedures showed that most of the artifacts were removed, with minimal EEG distortion (Allen et al., 2000). The EEG data were filtered by bandpass (1–50 Hz), re-referenced to the mean of the electrode TP 9 and TP 10. The GND channel was used a ground position on the MR compatible EEG cap. The severe noise or artifacts of the EEG data caused by environmental noise, muscular activity, eye activities and blinking were manually reduced to influences in the subsequent analysis.

### Analysis of Behavioral Data

For each of the three feedback conditions, Successful-Go (SG) and SS ratio were calculated to verify if the performance of each participant demonstrated typical inhibition functions (Verbruggen and Logan, 2009). The behavioral results of the performance in the stop signal task consisted of the reaction time in go trial (Go-RT) and pairwise comparison of cSSD among no-feedback, reward, and punishment conditions. The SSRT as defined in the horse race model (Logan et al., 2014) was calculated to characterize the response inhibition. Since the stopping process itself cannot observed directly, the SSRT was calculated by deducting SSD from the go RT. The response inhibition function was calculated as the ratio of the SS trials to the number of all stop-trials at the given SSDs. Inhibition functions of different feedback conditions were then subjected to a two-way Feedback Conditions (no-feedback, reward, and punishment) by SSD (cSSD, cSSD ± 40 ms, and cSSD ± 80 ms) within-subject ANOVA. *Post hoc* comparisons were carried out with Fisher’s least significant difference test.

### Analysis of fMRI Data

The first-level fMRI analysis was performed for response inhibition validation under feedback conditions and the analysis has been performed via first and second level statistical analysis. The first-level statistical analysis for each participant was conducted with a general linear model which include predictors that convolved a canonical hemodynamic response function (i.e., the block function in 3dDeconvolve of AFNI) with the onset of go stimulus in the SS and successful go (SG) conditions. Stimulus types and participants’ response conjointly determined the successful-go (SG), SS, and FS trials for each reward condition. The active regions of the brain for response inhibition were identified through the contrast between SS, and successful go (SG) conditions. The effect of inhibitory control was defined as the difference between SS and SG under no-feedback, reward, and punishment conditions. For the second-level analysis, the difference between the conditions (SS and SG) was examined with a linear mixed-effect model (3dMEMA). The whole brain Type I error controlled with a cluster alpha of 0.05 via Monte Carlo simulation (3dClustSim).

The second-level fMRI analysis was accomplished for the difference between the feedback conditions. The active regions of the brain for reward were identified through the contrast between reward and no-feedback conditions and that for punishment were identified through the contrast between punishment and no-feedback conditions. Thus, the effect of reward was defined as the difference between reward and no-feedback conditions, while the punishment was defined as the difference between punishment and no-feedback conditions. The difference between each feedback condition was tested with a 3dMEMA. The whole brain Type I error controlled with a cluster alpha of 0.05 via Monte Carlo simulation (3dClustSim). The current study carried out simultaneous fMRI-EEG recordings in order to capture both the rapid brain dynamics and precise spatial loci of the inhibitory process. fMRI results were then adopted to inform the selection of EEG region of interests (Huster et al., 2012) in this study.

### Analysis of EEG Data

**FIGURE 2:**
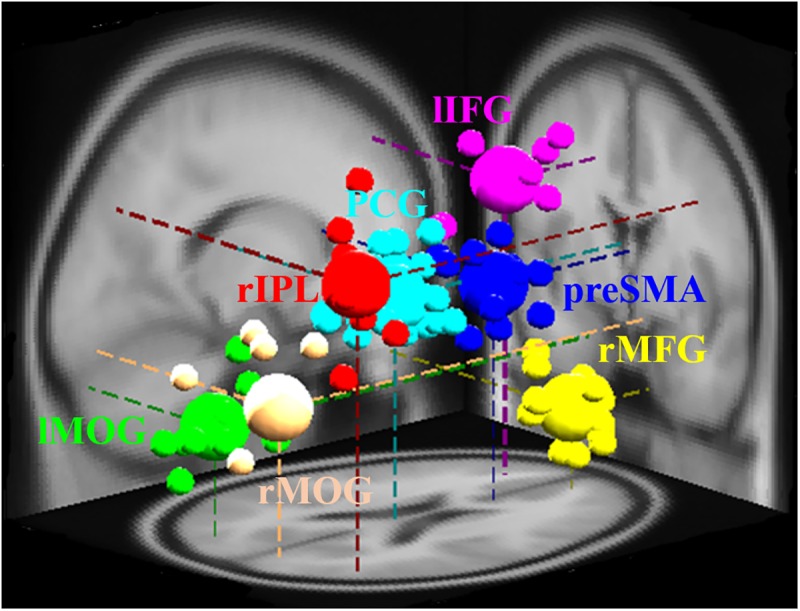
Different brain regions and related dipole source locations. Pre-SMA, pre-supplementary motor area; rMFG, right middle frontal gyrus; lIFG, left inferior frontal gyrus; PCG, posterior cingulate gyrus; rIPL, right inferior parietal lobe; lMOG, left middle occipital gyrus; rMOG, right middle occipital gyrus. Pre-SMA, lIFG, and rMFG are accomplished for response inhibition. PCG and rIPL are accomplished for emotion and motivation. Bilateral MOGs are accomplished for the analysis of visual stimuli. The small area shows the location of each participant’s dipole, while vast areas display the dipole locations of each cluster.

The study of EEG signal was performed in EEG-Lab. The independent component analysis method was carried out to separate the temporally independent time sequence of stimulation from which the position of the dipole source was located in the brain of each participant for cross-participant analysis or group analysis (Makeig et al., 1996; Oostenveld and Oostendorp, 2002). We extract the components of the artifact manually, and after that, we investigate the grouping of components based on K-means standards (statistics toolbox) and dipole-fitting coordinates to identify the best cluster of components. One of the seven resulting clusters was excluded because they were found in less than 75% of participants. The remaining six groups [pre-SMA, lIFG, right middle frontal gyrus (rMFG), right inferior parietal lobe (rIPL), posterior cingulate gyrus (PCG), and bilateral middle occipital gyrus (MOG)] and their dipolar source locations were recognized as presented in **Figure 2**. The baseline EEG signal was presented between -0.5 and 0 s before go events for matching of reaction magnitudes of consistent trials. Two conditions, specifically SS and SG, were recognized as a result of interest. A two-way ANOVA (conditions × scenarios) was performed on the EEG baseline dataset to confirm whether they are same through conditions and scenarios. To examine the changes in brain activity after the go stimuli and subsequent stop stimuli, each period (i.e., epoch) was independently converted into the time domain and frequency by event-related spectral perturbation (ERSP) analysis (Delorme and Makeig, 2004).

## Results

### Behavioral Results

**FIGURE 3:**
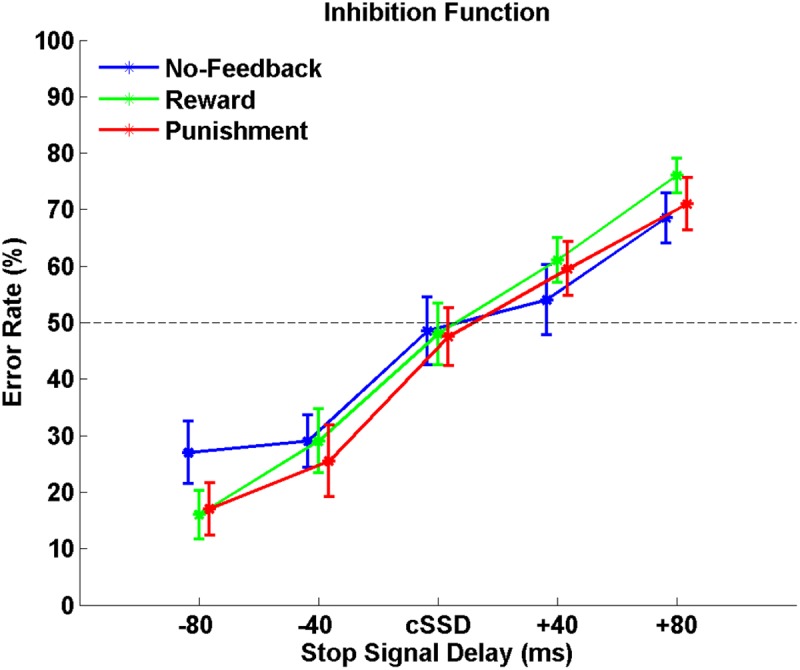
The response inhibition functions for no-feedback (blue), reward (green), and punishment (red) conditions. Error rates are plotted against five different SSDs centered around the cSSD that was adaptively determined for each individual participant and each condition, respectively. Error bars indicate standard error of the mean.

**Table 1** shows behavioral results in Go trials and Stop trials, including conditional means for Go RT, Go success rates, SSRT, cSSD, and SS rates. Only SG ratio and cSSD showed significant difference among feedback conditions [*F*(2,19) = 5.71, *p* < 0.05 and *F*(2,19) = 9.65, *p* < 0.05, feedback conditions main effect for SG ratio and cSSD, respectively]. *Post hoc* comparison showed that, for the SG ratio, we observed that both reward (84.9%) and punishment (85.0%) conditions had significantly higher SG ratios than the no-feedback (76.7%) condition (both *p*s < 0.01). In the Stop trials, we found that cSSD is significantly longer in the no-feedback condition (182 ms) than in the reward (177 ms; *p* < 0.05) and punishment (176 ms; *p* < 0.05) conditions. Because cSSD was determined prior to the formal experiment, the equivalent Stop success rate across conditions indicate the validity of the adaptive procedure. **Figure 3** shows the inhibitory functions (SS rate as a function of SSD) for the three feedback conditions, respectively. The two-way interaction between SSD and feedback condition was not significant [*F*(2,19) = 0.015, *p* > 0.05], corroborating the mean cSSD results that participants achieved similar level of performance in inhibitory control across different feedback conditions.

**Table 1:** Behavioral results under no-feedback, reward, and punishment conditions.

Trial types	Behavioral outcomes	No-feedback (*SD*)	Reward (*SD*)	Punishment (*SD*)
Go trials (75%)	Go-RT (ms)	420 ± 64	421 ± 38	424 ± 44
	SG ratio (%)	76.7 ± 11.2^∗^	84.9 ± 7.7	85.0 ± 5.9
Stop trials (25%)	cSSD (ms)	182 ± 60^∗^	177 ± 62	176 ± 57
	SSRT (ms)	238 ± 58	244 ± 49	248 ± 52
	SS ratio (%)	54.6 ± 19.3	54.0 ± 15.6	55.9 ± 18.9

*SG, successful go; SS, successful stop; SD, standard deviation. Asterisks indicate significance in t-tests pairwise between the denoted condition and the other feedback conditions.*

**FIGURE 4:**
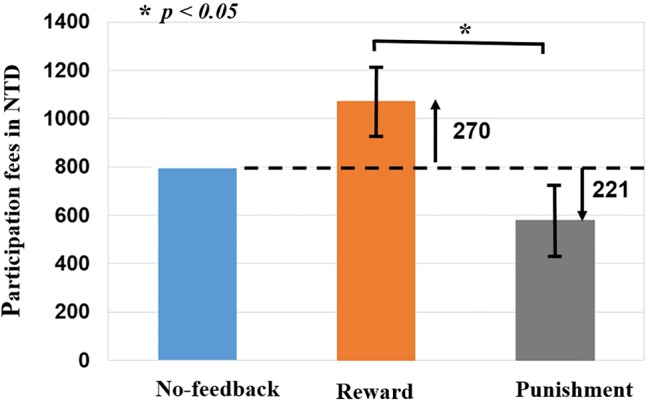
Difference of monetary fees in no-feedback, reward, and punishment conditions. Reward (i.e., positive feedback) shows greater fees then punishment (i.e., negative feedback), reward (+270 NTD) > Punishment (–221 NTD) fees. Asterisks indicate pairwise significance (*p* < 0.05) in *t*-tests between the no-feedback and reward conditions.

To verify the validity of monetary feedback, the each participant earned 270 NTD in the reward condition, and lost 221 NTD in the punishment condition. A greater difference in the absolute aggregated reward (270 NTD) than punishment (221 NTD, *p* < 0.05; **Figure 4** and **Table 2**), which resulted in a final average credit of 849 NTD. These results confirmed that overall one did receive positive performance feedback in the reward block and negative feedback in the punishment block.

**Table 2:** Difference of monetary fees in each participant under no-feedback, reward, and punishment conditions.

Participant ID	No- feedback	Reward	Punishment	Total payment amount (NTD)
1	800	+220	-180	840
2	800	+180	-340	640
3	800	+160	-330	630
4	800	+310	-250	860
5	800	+80	-400	480
6	800	+290	-140	950
7	800	+320	-260	860
8	800	+280	-120	960
9	800	+370	-130	1040
10	800	+210	-300	710
11	800	+290	-310	780
12	800	+310	-180	930
13	800	+360	-160	1000
14	800	+320	-110	1010
15	800	+180	-390	590
16	800	+250	-170	880
17	800	+350	-130	1020
18	800	+240	-200	840
19	800	+360	-130	1030
20	800	+320	-180	940
Average ± SD	800	+270 ± 78.13	-221 ± 94.28	849 ± 163

*In reward, the participants earned more money than the punishment condition*.

### Neuroimaging Results

#### fMRI Results of Response Inhibition

##### Response inhibition within each feedback condition

**FIGURE 5:**
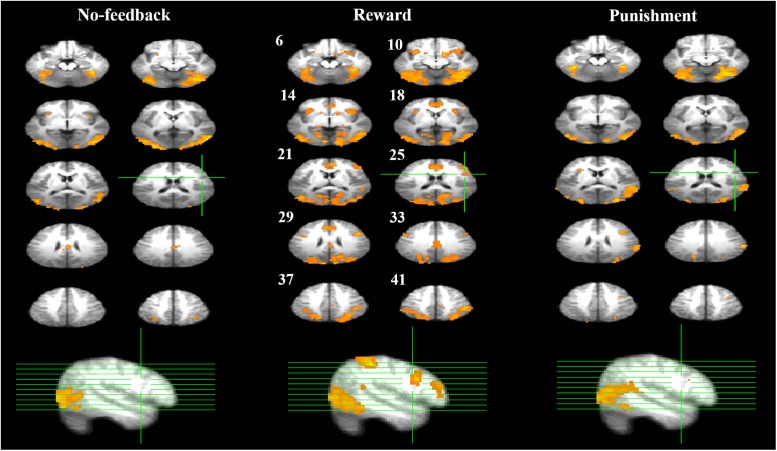
Response inhibition associated brain regions. Brain activations associated with the no-feedback (left panel), reward (middle panel), and punishment (right panel) are rendered on the axial slices ranging between 6 and 41 mm along the *z*-axis (spacing 4 mm between slices). The upper-left number next to each central slice indicates the *z*-axis, and the same set of slices are illustrated for all conditions. The right hemisphere is on the right side of the figure. The cluster threshold alpha was set at 0.01, and the voxel-wise threshold was fixed at *p* < 0.001.

**Table 3** presents the brain areas that showed significant response inhibition component (SS > SG) under the no-feedback, reward, and punishment conditions, respectively. **Figure 5** also shows these activations in these three conditions and their overlapping regions. In the no-feedback state, the left MOG (lMOG), lIFG, right fusiform gyrus (FG), right cingulate gyrus, right insula gyrus (IG), and a few different areas of the upper parietal lobe were activated (**Table 3A** and **Figure 5**, left panel). In the reward condition, the activated regions of the brain included right FG, right subcallosal gyrus (SCG), right supper temporal lobe, bilateral parahippocampal gyrus, PCG, and a few different regions in the frontal gyrus (**Table 3B** and **Figure 5**, middle panel). In the punishment condition, right MOG (rMOG), left FG, left supper temporal gyrus, left precuneus gyrus (PG), left gyrus of the insula (IG), and two areas of the rMFG showed significant activation (**Table 3C** and **Figure 5**, right panel).

**Table 3:** The activation of different brain regions under (A) no-feedback, (B) reward, and (C) punishment conditions.

Side	Brain areas	BA	MNI coordinate (mm)			Cluster size (voxels)
			*X*	*Y*	*Z*	
**(A) SS > SG in no-feedback condition**					
R	Fusiform gyrus	37	-48	60	-18	1152
L	Middle occipital gyrus	19	36	78	-15	776
R	Cingulate gyrus	23	-6	18	33	76
R	Superior parietal lobule	7	-36	69	57	70
L	Superior parietal lobule	7	33	63	54	30
L	Inferior frontal gyrus	47	33	-21	-6	24
R	Insula gyrus	13	-36	-18	0	21
**(B) SS > SG in reward condition**					
R	Fusiform gyrus	37	-42	45	-21	5497
R	Middle frontal gyrus	10	-9	-48	15	480
R	Subcallosal gyrus	–	-15	-12	-12	363
L	Inferior frontal gyrus	47	33	-21	-12	165
R	Middle frontal gyrus	46	-51	-39	21	136
L	Cingulate gyrus	23	0	15	33	134
R	Inferior frontal gyrus	9	-51	-15	27	104
L	Inferior frontal gyrus	9	45	-6	30	57
R	Superior temporal gyrus	22	-57	42	12	38
R	Middle frontal gyrus	6	-30	-15	63	27
R	Parahippocampal gyrus	27	-27	27	-6	24
L	Parahippocampal gyrus	27	21	30	-6	20
**(C) SS > SG in punishment condition**					
R	Middle occipital gyrus	19	-33	87	12	1623
L	Fusiform gyrus	37	39	45	-21	895
R	Middle frontal gyrus	9	-42	-12	30	39
L	Superior temporal gyrus	40	51	48	21	36
L	Precuneus gyrus	7	18	72	36	33
L	Insula gyrus	13	30	-21	12	24
R	Middle frontal gyrus	8	-42	-6	45	21

*Voxelwise threshold, p = 0.001; alpha cluster < 0.01; BA, Brodmann area; R, right; L, left*.

##### Contrasting inhibitory control among feedback conditions

**FIGURE 6:**
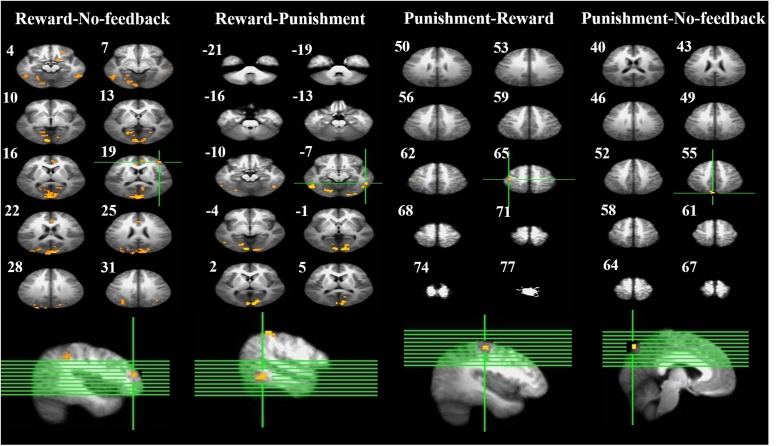
Brain activation related to response inhibition. Left-panel: horizontal sections under reward–no-feedback; middle-panels: horizontal slices under reward–punishment and punishment–reward; right-panel: horizontal slices during the punishment–no-feedback condition. The upper left-hand number in addition to each segment indicates the *z*-axis. The voxel-wise threshold statistics were set at *p* < 0.001, and cluster threshold alpha <0.01. The right-hemisphere is on the right side of the picture.

**FIGURE 7:**
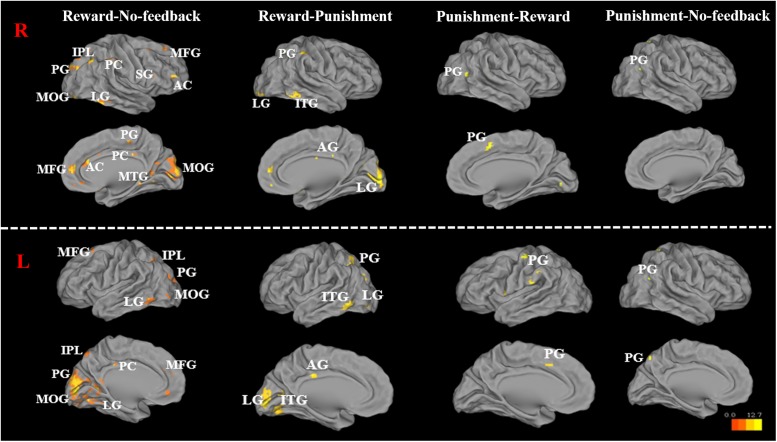
Significant brain activation in the cortical regions, for reward–no-feedback, reward–punishment, the punishment–reward, and punishment–no-feedback condition under response inhibition (R, right hemisphere in the top two rows; L, left hemisphere in the bottom two rows). Please see **Table 4** for the abbreviation of all brain regions.

To better quantify differences in inhibitory control among the three feedback conditions, we further contrasted inhibitory control for “reward–no-feedback” and “punishment–no-feedback” (**Table 4** and **Figures 6**, **7**). Significant activation in the “reward–no-feedback” contrast (**Table 4A** and **Figures 6**, **7**, left panels) encompassed a widely distributed network including the left lingual gyrus, left PG, left middle temporal gyrus (MTG), left anterior cingulate gyrus, left inferior semi-lunar lobule, lMOG, rIPL, right postcentral gyrus, right inferior temporal gyrus, right posterior cingulate, and a few different regions in the rMFG. On the other hand, the “punishment–no-feedback” contrast only revealed significant activation in the left PG (**Table 4D** and **Figures 6**, **7**, right panels). Interested readers can also find results of “reward–punishment” and “punishment–reward” contrasts in the Supplementary Materials for more intensified differences between conditions. These significantly activated brain regions guided the analysis of the temporal oscillation of EEG clusters associated with feedback described below.

**Table 4:** Significant activation in SS compared with SG under (A) reward–no-feedback, (B) reward–punishment, (C) punishment–reward, and (D) punishment–no-feedback contrast.

Side	Brain areas	BA	MNI coordinate (mm)			Cluster size (voxels)
			*X*	*Y*	*Z*	
**(A) Inhibitory control (SS > SG) in reward–no-feedback under BFS**					
L	Lingual gyrus	17	6	87	-3	585
L	Precuneus gyrus	7	30	66	42	188
R	Inferior parietal lobule	7	-39	66	48	130
L	Lingual gyrus	18	21	60	3	72
L	Middle temporal gyrus	37	51	51	-9	61
R	Postcentral gyrus	40	-54	33	51	55
R	Inferior temporal gyrus	37	-57	54	-15	48
R	Posterior cingulate	30	-12	57	12	45
L	Anterior cingulate	24	0	-27	27	33
R	Middle frontal gyrus	10	-9	-48	15	32
R	Middle frontal gyrus	11	-33	-36	-18	27
L	Inferior semi-lunar lobule	–	21	81	-51	26
R	Middle frontal gyrus	10	-45	-45	15	25
R	Middle frontal gyrus	8	-30	-12	63	24
R	Middle frontal gyrus	25	-15	-12	-18	23
L	Middle occipital gyrus	18	24	81	18	23
**(B) Inhibitory control (SS > SG) in reward–punishment under BFS**					
L	Lingual gyrus	17	6	90	-3	324
R	Inferior parietal lobule	40	-54	39	57	86
L	Precuneus gyrus	19	30	66	39	69
R	Superior parietal lobule	7	-33	69	48	66
L	Lingual gyrus	18	24	75	-12	65
L	Inferior temporal gyrus	37	54	60	-12	61
R	Middle temporal gyrus	20	-57	45	-12	45
L	Angular gyrus	39	36	78	33	31
**(C) Inhibitory control (SS > SG) in punishment–reward under BFS**					
L	Precentral gyrus	4	39	21	60	20
**(D) Inhibitory control (SS > SG) in punishment–no-feedback under BFS**					
L	Precuneus gyrus	7	3	81	51	22

*Voxelwise threshold, p = 0.001; alpha cluster < 0.01; BA, Brodmann area; R, right; L, left*.

### EEG Results of Response Inhibition

**Figure 2** presents the seven groups (rMFG, pre-SMA, lIFG, PCG, rIPL, and bilateral MOGs) and their dipoles sources location used to measure the EEG activities during no-feedback, reward, and punishment conditions under response inhibition or inhibitory control. Accordingly, the rMFG is measured to be a crucial area for maintaining attention rather than stopping the action. Therefore, the rMFG is considered as an essential region for maintaining attention instead of stopping the action. On the other hand, pre-SMA and lIFG are considered as directly associated with the inhibitory control. However, pre-SMA, lIFG, and rMFG were implicated at the stage where sustained attention and response inhibition was assumed to occur. Additionally, the PCG has been widely considered to be related to emotional salience. The rIPL has also been linked to the perception of emotions in facial stimuli. However, bilateral MOGs were considered generally related to the visual perception that is less specifically related to the stop signal task. The ERSP powers were examined in visual processing time and presented in Supplementary Figures 3, 4.

**FIGURE 8:**
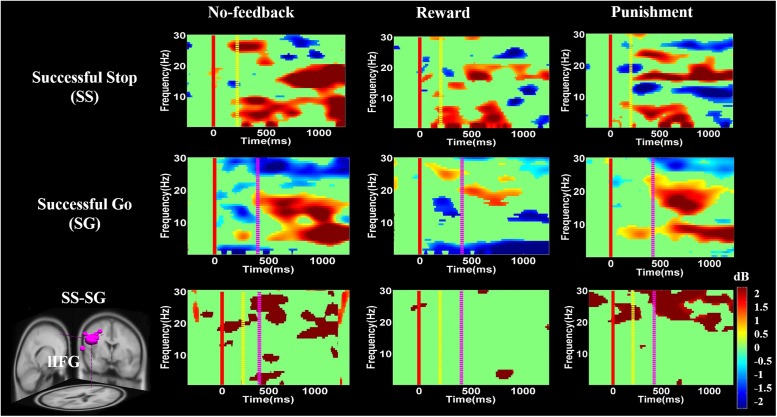
The event-related spectral perturbation (ERSP) of the lIFG cluster during response inhibition. The solid red line shows go-stimulus onset. Yellow dashed line reveals stop signal onset. Purple dashed line presents response onset. Statistic at *p* < 0.01. Color bars indicate the scale of ERSP.

**FIGURE 9:**
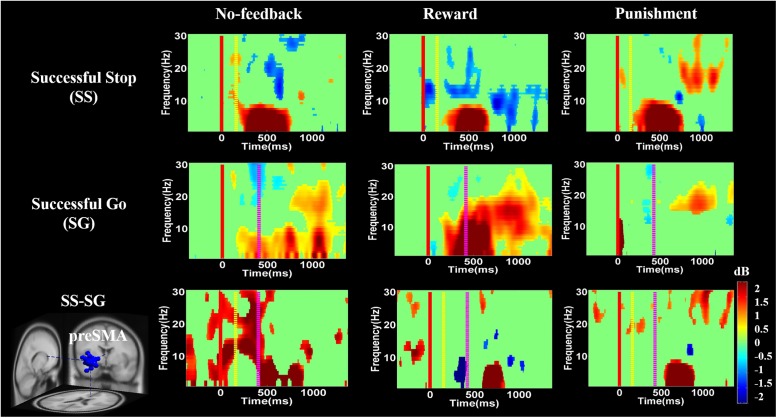
The ERSP analysis in the pre-SMA of the brain during response inhibition. Solid red line shows go cue onset. Yellow dashed line displays stop signal onset. Purple dashed line presents response cue onset. Statistic at *p* < 0.01. Color bars reveal the level of ERSP.

**FIGURE 10:**
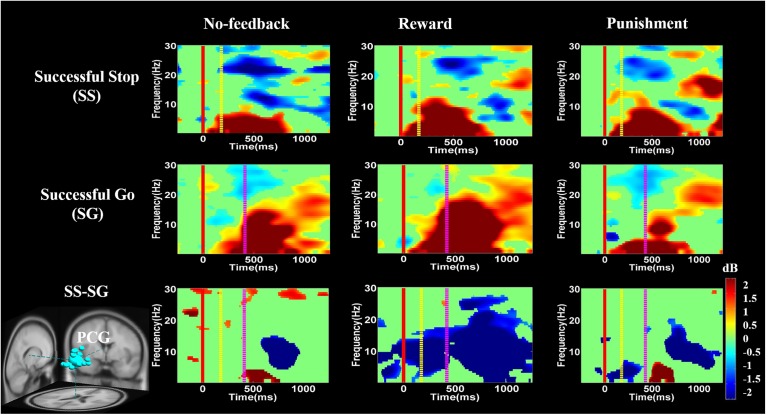
The ERSP analysis in the PCG of the brain during response inhibition. Solid red line shows go stimulus onset. Yellow dashed line reveals stop signal onset. Purple dashed line presents response onset. Statistic at *p* < 0.01. Color bars indicate the magnitude of ERSP.

**FIGURE 11:**
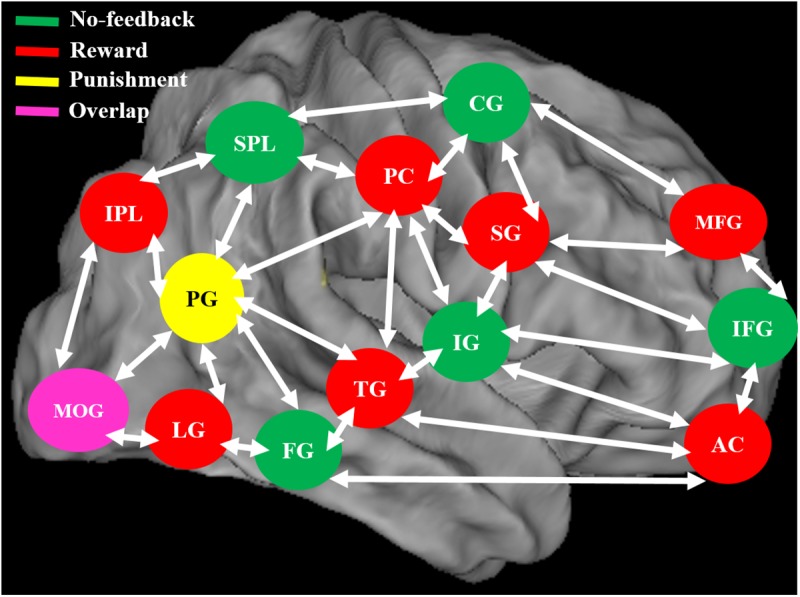
The neural system shows the different brain activation and overlap area of the brain during no-feedback, reward, and punishment conditions under human inhibition. An overlap middle occipital gyrus (MOG) of the brain was investigated in three conditions.

For the results of lIFG, pre-SMA, PCG, and rIPL, the emphasis is placed on contrasts for inhibition of response through contrasting SS vs. SG in each condition. Significant brain oscillations can be found in each condition (i.e., no-feedback, reward, and punishment) in **Figures 8**–**11**, which are mainly explained in the following paragraphs. The baseline power of the EEG signals was assumed to be equal concerning the SS and SG contrasts under these situations since the participants had to be in a similar state before the demonstration of the stimulus in each state. Consider with this hypothesis, one-way ANOVA comparing SS and SG in the conditions of no-feedback, reward, and punishment did not observe any significant difference. Baseline power results are shown in Supplementary Figures 5–11.

To ensure the equivalence of baseline power among feedback conditions within each participant as they prepared to inhibit their response in each trial, we applied trial-by-trial one-way ANOVAs to compare the differential spectrum of SS–SG during a 500 ms baseline period across each feedback condition and only examined trials showing no significant differences among conditions during the baseline period (see Supplementary Figures 5–11 for details).

**Figures 8**–**10** show the results of ERSP analyses in lIFG, pre-SMA, PCG, rIPL. **Figure 8** displays high synchronization of delta and theta band powers in reward than in the no-feedback and punishment conditions during a SS in the lIFG. Additionally, high synchronization of beta band power was examined in reward than in the no-feedback and punishment conditions during successful go in the lIFG. Moreover, in the “SS vs. SG” contrast, high synchronization of beta band activity was observed in the no-feedback and punishment conditions compared to the reward condition in the lIFG. **Figure 9** shows higher synchronization of the delta, theta, and alpha bands were investigated in no-feedback and punishment conditions than in reward condition during SS trials in the pre-SMA. Moreover, higher synchronization of delta, theta, and alpha bands was examined during reward than in the no-feedback and punishment conditions under successful go trials in the pre-SMA. Additionally, in the SS–SG contrast, desynchronization of delta, theta, and alpha bands was observed during reward than in the no-feedback and punishment conditions in the pre-SMA.

**Figure 10** presents high synchronization of the delta, theta, and alpha band powers in reward than in the no-feedback and punishment conditions during SS and SG circumstances in the PCG. Furthermore, in the SS–SG contrast, desynchronization of theta–alpha activities was observed during reward than in the no-feedback and punishment conditions. Supplementary Figure 1 shows synchronization of delta band power in reward and punishment than in no-feedback condition during SS and SG trials in the rIPL. Additionally, desynchronization of alpha and beta activities were observed in reward than in the no-feedback and punishment conditions during the SS–SG contrast in the rIPL. The ERSP analysis in rMFG, lMOG, and rMOG are described in the Supplementary materials (Supplementary Figures 2–4).

## Discussion

The primary purpose of this study is to compare the neural mechanism of response inhibition under no-feedback, reward, and punishment conditions in battlefield settings. In terms of behavioral performance, we found significant difference among feedback conditions in SG ratio (reward and punishment higher than no-feedback) and cSSD (reward and punishment shorter than no-feedback), indicating that the adaptive monetary incentive modulated the participants’ performance under response inhibition. In terms of fMRI results, distinct regions showed preference per the three feedback conditions (no-feedback condition: right FG, left pre-SMA, lIFG, bilateral SPL, lMOG, and right IG; the reward condition: left LG, rIPL, left PG, right SPL, left ITG, right MTG, left AG; the punishment condition: the left PG. Finally, regarding EEG results, we found positive feedback related stronger changes of the delta, theta, and alpha synchronization in the PCG than that of the negative or no-feedback conditions.

First of all, activation regardless of feedback verified that our task has effectively engaged the response inhibition network. Consistent with what was found after the stop signal overall in the current study, Menon and Uddin (2010) reported that the IG is involved in cognitive control and attentional processes. Cai and Leung (2011) used the stop signal task with fMRI analysis and found that the inferior frontal cortex (IFC) and the anterior insula are more active during the inhibition of response. Moreover, as the MOG is involved in the visual perception and recognition of the stimulus shape (Grill-Spector and Malach, 2004), and the parietal region plays an essential role in visual attention and spatial orientation (Yantis et al., 2002), participants may have engaged more visual and attentional resources to follow the stop stimulus and inhibit their response as soon as possible. The pre-SMA and right IFG activation have also been demonstrated for the contrast among SS and successful-go (Swick et al., 2011; Ko et al., 2016). Secondly, we also found higher PCG activation in the reward condition than in the no-feedback condition, indicating that the reward may induce higher emotional response, and may thus require stronger inhibition to suppress the response (Enzi et al., 2015).

When contrasting the response inhibition component in these three conditions, the significant brain regions were found showing higher brain activation for the reward via reward–no-feedback contrast (see **Table 4** and **Figure 11**). Activated left PG was found in the punishment–no-feedback contrast, this finding is consistent with previous studies on the effect of punishment (Menon et al., 2001; Makiko et al., 2012). Specifically, Menon et al. (2001) examined error-related brain activity associated with failure to inhibit response during a go/no-go task and found error-related brain activation in the left precuneus and PCG. Moreover, Makiko et al. (2012) reported that when mitigating criminal sentences (punishment), precuneus and anterior cingulate cortex showed stronger activation. In all feedback conditions, MOG was found to be activated for the contrast of response inhibition (see **Figure 11**). Another way to examine the effect of monetary feedback on response inhibition in the current study is via the direct contrasts of “reward–punishment” and “punishment–reward.” Because the results are essentially similar to what has been described, interested readers can find the results of these contrasts in the supplementary materials (Supplementary Figures 1–4).

The response inhibition contrast also identified the right-temporoparietal junction (rTPJ), which has been implicated, together with the right inferior parietal lobule, in identifying behaviorally salient events (Husain and Nachev, 2007). Also, Chang et al. (2013) used transcranial magnetic stimulation to interfere with bilateral rTPJ to investigate the role of attention networks and to observe that rTPJ seriously participates in attention reorientation. In addition, the right TPJ is also engaged in the system of “Theory Of Mind” (TOM) which recruits the medial-prefrontal gyrus, right-superior temporal sulcus, precuneus, and bilateral TPJ (Saxe and Kanwisher, 2003; Aichhorn et al., 2009). The TOM network showed stronger metabolic activities when thinking about others’ feelings. On the other hand, Koster-Hale et al. (2013) used the analysis of multiple voxel patterns to observe the variance between intentional and unintentional damages to other people and to make the right TPJ related to moral judgments. The present study also replicates the result that, at least in no-feedback conditions, right TPJ may serve one or a few of these functions revealed above because the task involve gunfire decision which may aim at innocent hostage.

Previous studies have linked processing of rewards and punishments to activations in the IFG and anterior insula (Knutson et al., 2001; Haber and Knutson, 2010). Somewhat similar to these previous findings, the current fMRI study has found that the frontal gyrus and the anterior cingulate gyrus played a significant function under reward processing. Significant brain activation during reward processing has been observed in the lateral prefrontal cortex, hippocampus, and thalamus (Haber and Knutson, 2010). Previous investigations have reported that inferior frontal lobe, a major part of the lateral prefrontal brain area, is related to cognitive and emotional processing (Petrides, 2005). Ray and Zald (2012) have also shown that lateral PFC is involved in the regulation of emotion. All these studies revealed rewards processing in the brain that is similar to the current results.

Rosell-Negre et al. (2014) have shown that during the inhibition of response under the reward processing, frontal, parietal, and subcortical regions showed enhanced brain activation. During both reward and punishment situation, different brain regions demonstrated higher activation in left inferior frontal, precentral, right inferior occipital gyrus, and middle temporal lobe (Shigemune et al., 2014). Reward process may alter emotional and motivational states, and thus the participants’ impulses for making a shooting response could be greater than those in no-feedback and punishment conditions. The brain regions related to response inhibition may boost the level of activation to a greater extent in canceling the prepotent response under reward situation as compared to no-feedback and punishment conditions. To verify this assumption with higher sensitivity, we localized seven brain regions for no-feedback, reward, and punishment condition through the SS–SG contrast to the outcome of the condition, including rMFG, pre-SMA, lIFG, PCG, rIPL, and bilateral MOGs. Among these regions, the PCG, rIPL, and bilateral MOGs show greater activations in the reward condition. The PCG is strongly related to the emotional salience and executive function. The rIPL is linked to the perception of the emotions in the facial stimuli, and the rIPL participates in the manipulation of tools and decision-making roles (Kübler et al., 2003; Ishibashi et al., 2011). The involvement of PCG and IPL regions suggest that when participants engage in the task of (virtual) shooting with a pistol (i.e., a tool) at a kidnapper or terrorist on the BFS.

One previous study used stop signal task with EEG analysis and demonstrated that the pre-SMA play a significant role in the control of motor action (Huster et al., 2013). These authors found hand movement related neural signatures such as delta–theta band powers increasing in pre-SMA except for the beta band activity (Huster et al., 2013). Pre-SMA is necessary for the translating volitional feelings to actions (Penfield and Welch, 1951; Fried et al., 1991). These aforementioned studies also reported stronger beta band power as a neural signature of explicit responses. The current study also found related neural signatures (i.e., delta–theta band powers) during 400–700 ms time window. These findings suggest that participants make the actual response to the go stimulus and inhibit response to the stop stimulus in no-feedback, reward, and punishment conditions. In addition, the ERD of the beta band activity was found before and during response and then the ERS would follow the real response (Schulz et al., 2014; Ko et al., 2016). In the present study, the ERD of the beta band activity was observed in SS and successful go conditions during no-feedback, reward, and punishment conditions, because participants have already prepared the response when they see go stimulus. However, the ERS of the beta band activity naturally happens in the successful go condition because participants do not carry out the actual response in the SS condition under these three conditions.

Moreover, ERSP analysis of the SS condition found that the delta, theta, and alpha band powers were found higher in the reward than in the punishment and no-feedback conditions. According to Huster et al. (2013), the burst theta band power in frontal region is related to successful response inhibition. The current study also found the synchronization of delta, theta, and alpha band powers in reward than in the punishment and no-feedback conditions under SS in the pre-SMA, suggesting that the impulsivity in the reward is stronger than in the punishment and no-feedback conditions. In addition, a greater synchronization of delta and theta band powers were found in reward than in the punishment and no-feedback conditions under SS condition in the lIFG. However, a greater synchronization of the beta band power was found in reward than in the punishment and no-feedback conditions under SG condition in the lIFG, suggesting that the neural oscillations in the reward condition is stronger than in the punishment and no-feedback conditions. These findings also suggested the motivational effects on participants’ performance under reward compare to punishment and no-feedback conditions.

Recently, Swann et al. (2012) have shown decreased theta and alpha band powers and increased beta band power in the right frontal gyrus under the SS condition. The beta band power of the right frontal gyrus can be used to calculate the consistency with pre-SMA. Thus, they suggested that the right frontal gyrus can detect the SS and after that transfer the information to pre-SMA via beta band activity. These findings demonstrate the function of right frontal gyrus in SS under no-feedback, reward, and punishment conditions. Accordingly, it was suggested that the rMFG is involved in the transfer of information, not directly related to response inhibition. Therefore, it seems that different conditions may evoke different spectral perturbations. The ERSP of these three conditions under bilateral MOG are similar, due to their similar functions in the processing of visual stimuli. In addition, the adaptive monetary reward enhanced the participants’ performance under response inhibition. Reward contingencies increased the activation of the brain regions involved in response inhibition processes including the lIFG and pre-SMA. The positive performance feedback elicited higher brain activation compared to negative performance feedback. Moreover, simultaneous fMRI and EEG study confirmed that the characterization of functional activations and frequency oscillations of brain networks are under the same experimental condition, and thus more likely the same neural networks (Mulert, 2013). Accordingly, in this study, we found a common activated brain region (i.e., PCG) in both fMRI and EEG recording. We observed stronger changes of delta, theta, and alpha synchronization in the PCG under positive feedback than in the negative or no-feedback conditions.

Finally, some limitations of the current study are worth mentioning: only 2 out of 20 participants were females, which may lead to problem in generalizing the result to both genders; the battle field scenario in the current study were from a well-known shooting game (Counter-Strike), which uses only 2D images and may not be the most realistic settings for our participants. Future work may construct even more realistic environment in virtual reality to enhance the immersiveness and increase the impact of feedback. The current study has not fused the analytical procedure of the simultaneously recorded fMRI and EEG data, which may further limit the brain regions and dynamics correspond to the interaction between monetary feedback and response inhibition.

## Conclusion

The present study examined the neural mechanism of response inhibition under reward and punishment with the simultaneous fMRI-EEG system, and the results indicated that positive and negative monetary feedback did make a difference in terms of both behavioral indices of inhibition and the corresponding brain activities. The higher brain activation in the reward than in the punishment and no-feedback conditions may reflect higher motivation to concentrate and thus more intense processing of response inhibition. The current findings signified the importance of the schemes of feedback on withholding responses in scenarios simulating response decision that may lead to critical consequences in real world. Future studies along the same line may benefit from testing participants from the military profession and see how experience may shape the interaction between response inhibition and feedback processing.

## Author Contributions

L-WK initialized the research idea and designed the experimental paradigm, advised the methods of EEG data analysis in the collection of fMRI-EEG data. RC performed the fMRI-EEG signals collection, data analysis and drafted the manuscript. EC provided advice on the design of the experiment and fMRI data analysis as well as manuscript revisions. C-TL, Y-CL, and D-SL provided more advices on the manuscript revisions. All authors agreed on the final paper submission.

## Conflict of Interest Statement

The authors declare that the research was conducted in the absence of any commercial or financial relationships that could be construed as a potential conflict of interest.
