# Supplementary material for: Monetary Reward and Punishment to Response Inhibition Modulate Activation and Synchronization Within the Inhibitory Brain Network
Source: Front Hum Neurosci. 2018 Mar 1;12:27. doi: 10.3389/fnhum.2018.00027 (PMC5837970; doi:10.3389/fnhum.2018.00027)
Supplement: Supplementary file 1 [file Data_Sheet_1.docx]

Supplementary Materials

**Monetary Reward and Punishment to Response Inhibition Modulate Activation and Synchronization within the Inhibitory Brain Network**

Rupesh Kumar Chikara^†^, Erik C. Chang^†^, Yi-Chen Lu, Dar-Shong Lin, Li-Wei Ko^*^

*** Correspondence:** Li-Wei Ko ([lwko@mail.nctu.edu.tw](mailto:lwko@mail.nctu.edu.tw))

^†^ Equal contributions

***Contrasting inhibitory control among feedback conditions***

In the “Reward-Punishment” contrast, the left lingual gyrus, left precuneus gyrus, left inferior temporal lobe, left angular gyrus, right inferior parietal lobe, right superior right parietal lobe, and right middle temporal gyrus were stimulated in this contrast (Table 4B, Figures 6 and 7 middle panel). Moreover, in the “Punishment – Reward” contrast revealed significantly higher activation merely in left precentral gyrus, as displayed in (Table 4C, Figures 6 and 7 middle panels).

Activated angular gyrus was found in (Reward – Punishment) contrast, which has been associated with visual attention and awareness. In addition, the angular gyrus might be involved in reorienting or shifting attention. For example, when the attention system is transformed to specific stimuli that have high salience in terms of movement, emotion. (Seghier, M. L. 2013). Accordingly, angular gyrus showed significant brain activation in attention and emotion.

***EEG Results of Response Inhibition***

Supplementary Figure 2 displays weak synchronization of the delta, theta-alpha bands were detected during reward than in the no-feedback and punishment conditions under successful stop (SS) condition in right MFG. Moreover, high synchronization of delta, theta-alpha and beta activities were found in the reward than in the no-feedback and punishment conditions during successful go (SG) condition in the right MFG. Furthermore, desynchronization of the delta, theta, alpha and beta activities were investigated in reward than in the no-feedback and punishment conditions during (SS-SG) contrast in the right MFG. However, rMFG activation was focused during the time in which constant attention and response inhibition was assumed to be occurring. Therefore, the rMFG area of the brain is measured as directly associated with response inhibition.

Supplementary Figure 3 displays the synchronization of theta and alpha band powers in reward compared to no-feedback and punishment conditions under SS and SG conditions in left MOG. Additionally, synchronization of theta-alpha bands was identified during reward than in the no-feedback and punishment conditions under (SS-SG) contrast in left MOG. Supplementary Figure 4 presents the synchronization of alpha band power in reward and punishment compared to the no-feedback condition under SS and SG conditions in right MOG. Moreover, synchronization of alpha activities was investigated in reward and punishment conditions then no-feedback condition during (SS-SG) contrast in right MOG. Left and right-MOGs were measured merely associated with a visual stimulus which was comparatively slight to the task of stop-signal. Their ERSP power was investigated in the visual processing period.

**
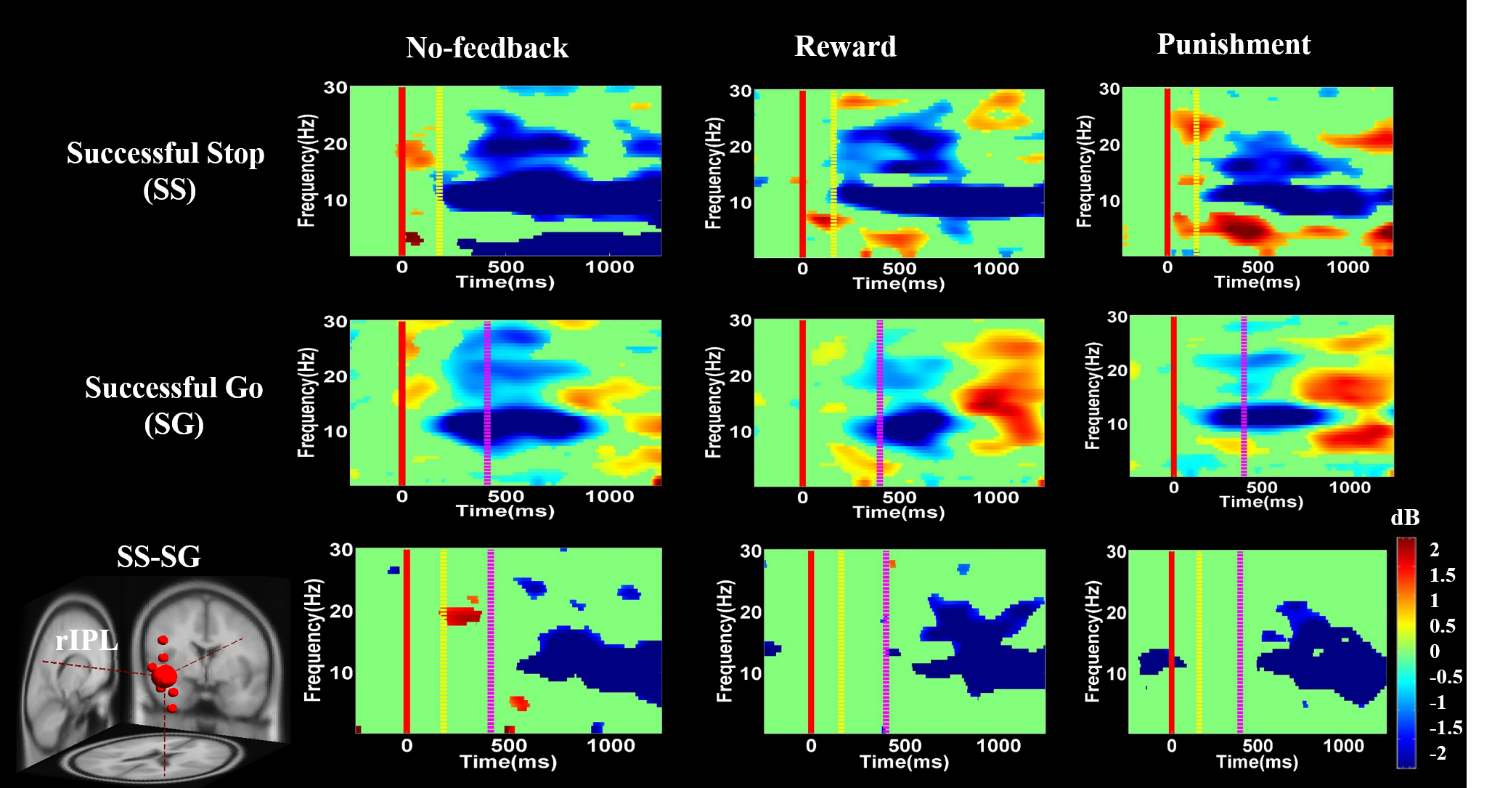
Supplementary Figure 1**: **The ERSP investigation in the right-IPL of the brain during response inhibition.** Solid red line shows go stimulus onset. Yellow dashed line presents stop signal onset. Purple dashed line reveals response onset. Statistic at p <0.01. Color bars indicate the scale of ERSP.

**
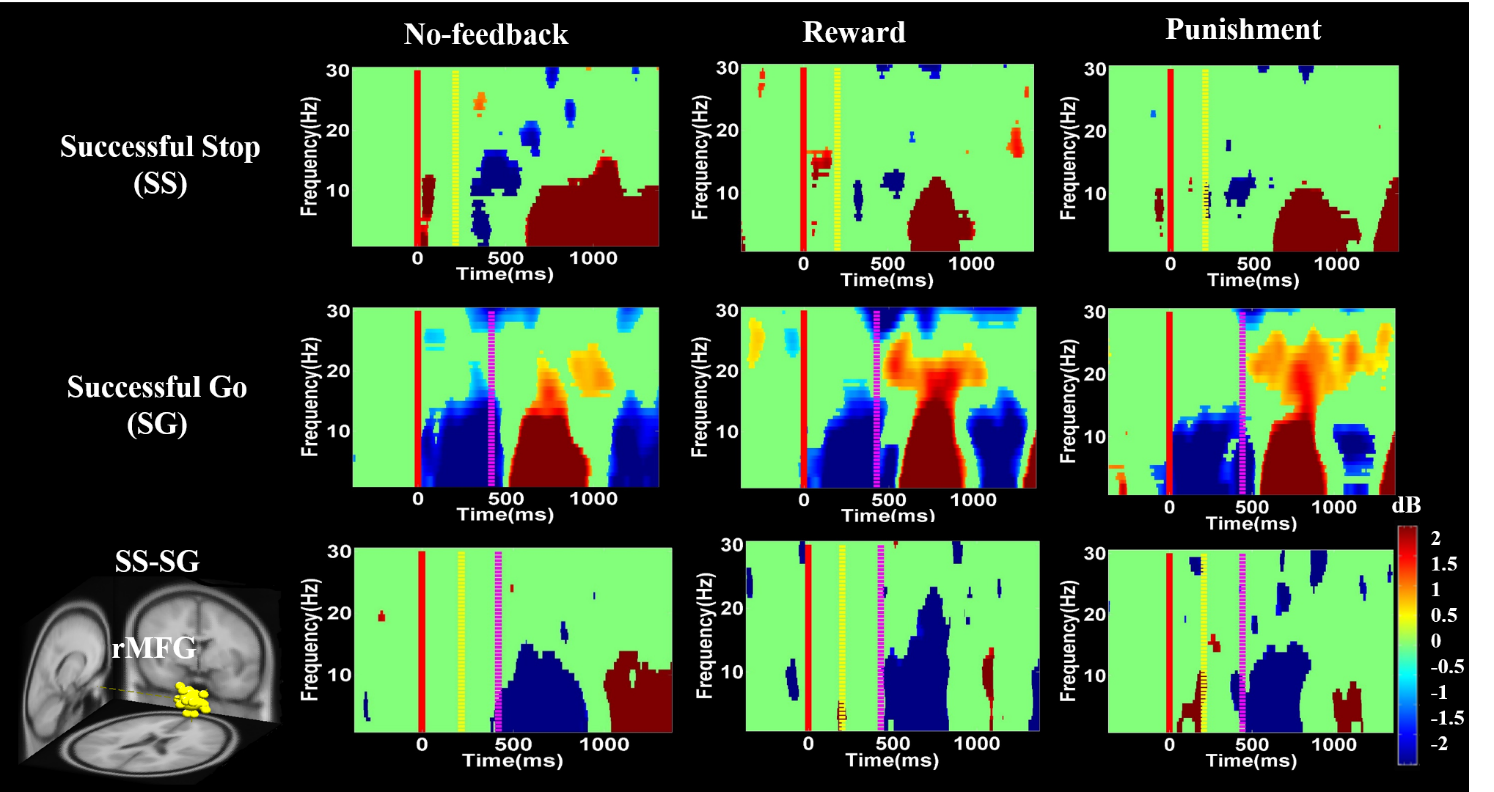
Supplementary Figure 2.** The event-related spectral perturbation (ERSP) images of right MFG cluster under inhibitory control. Red solid line: onset of the go stimulus; yellow dash line: onset of the stop signal; purple dash line: onset of response; color bars indicate the magnitude of the ERSPs; statistical threshold at p < 0.01.


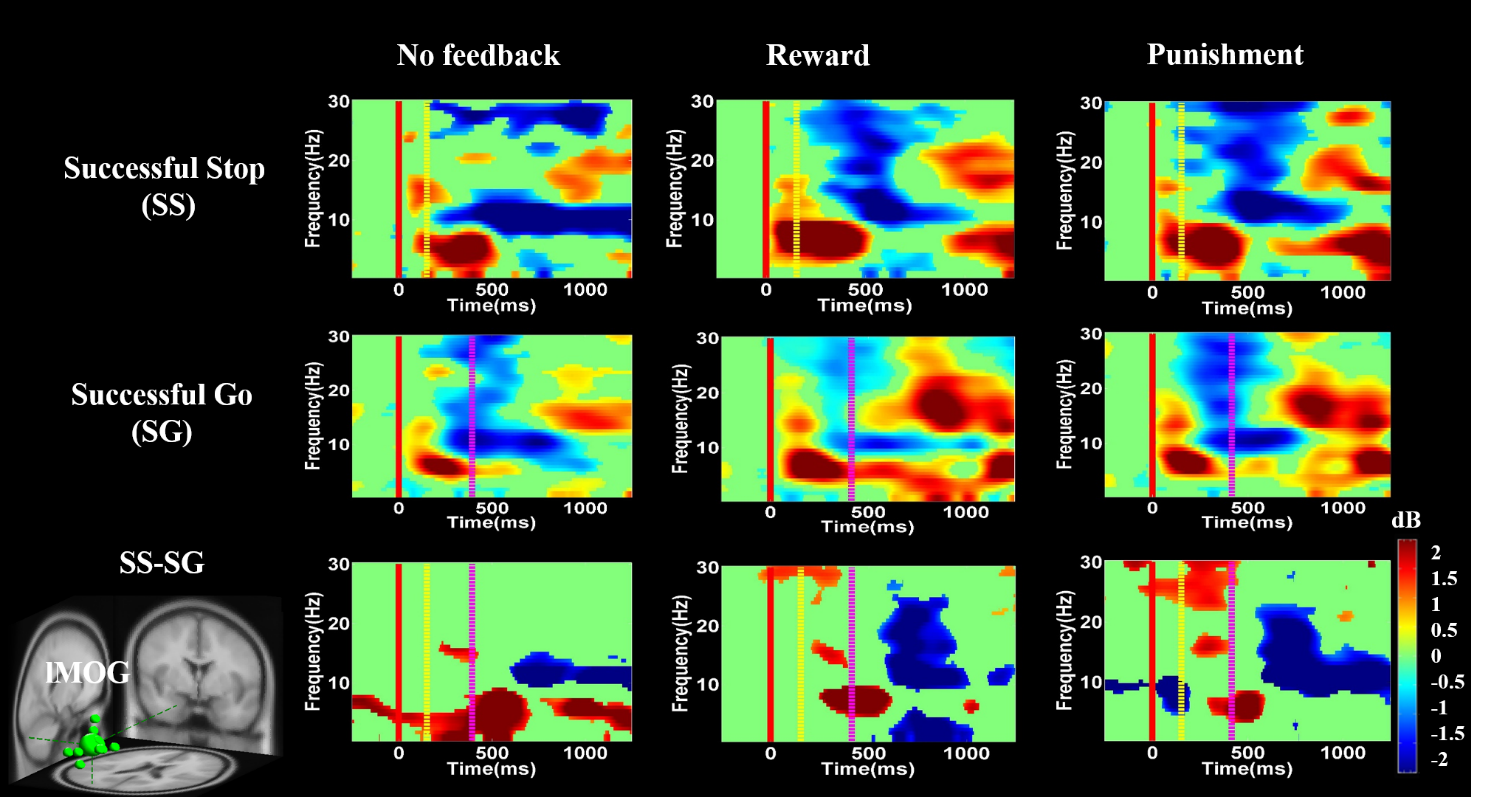
**Supplementary Figure 3.** The ERSP images of left MOG cluster for processing visual stimuli under inhibitory control. Red solid line: onset of the go stimulus; yellow dash line: onset of the stop signal; purple dash line: onset of response; color bars indicate the magnitude of the ERSPs; statistical threshold at p < 0.01.


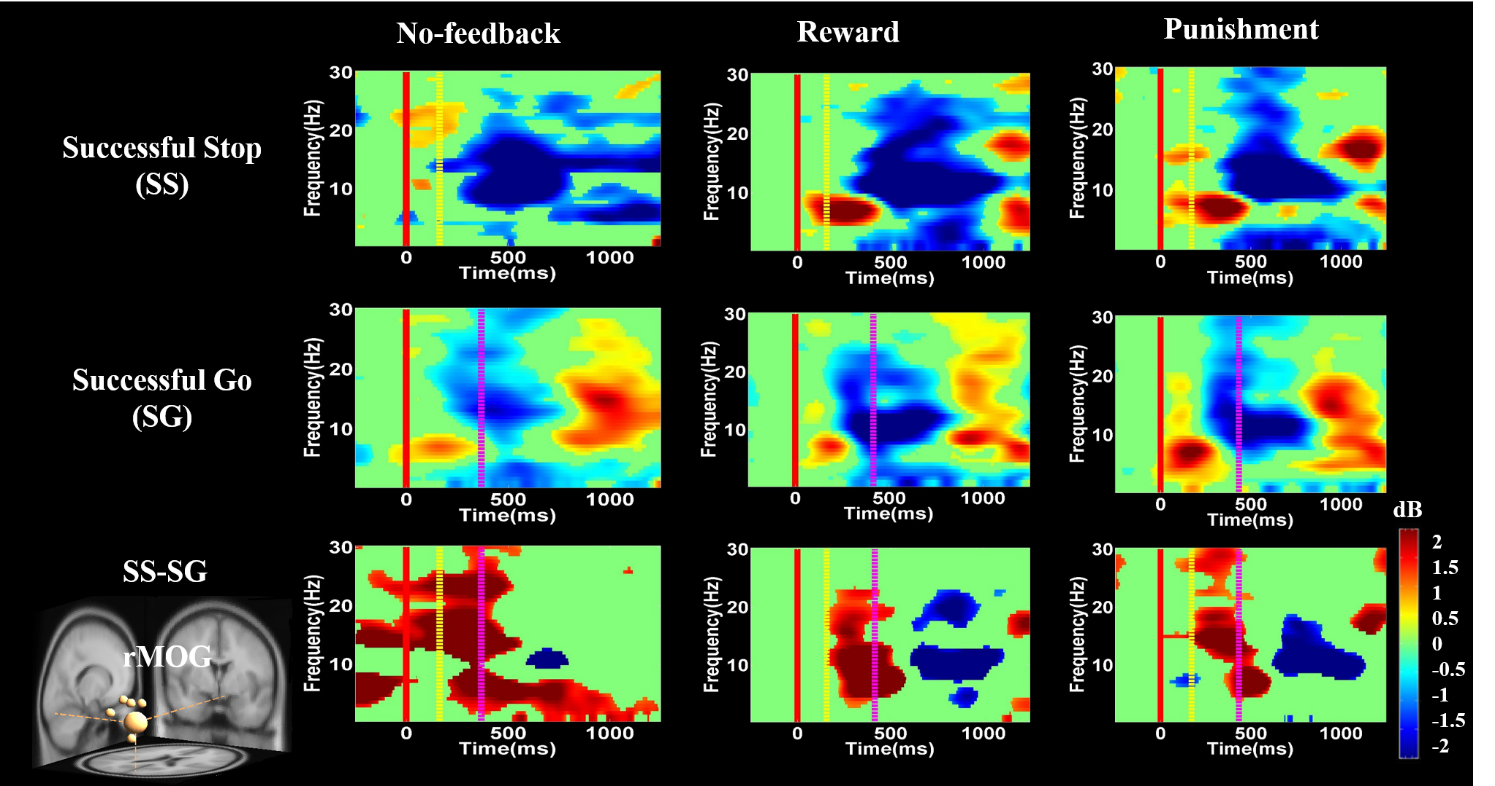
**Supplementary Figure 4.** The ERSP images of right MOG cluster for processing visual stimuli under inhibitory control. Red solid line: onset of the go stimulus; yellow dash line: onset of the stop signal; purple dash line: onset of response; color bars indicate the magnitude of the ERSPs; statistical threshold at p < 0.01.


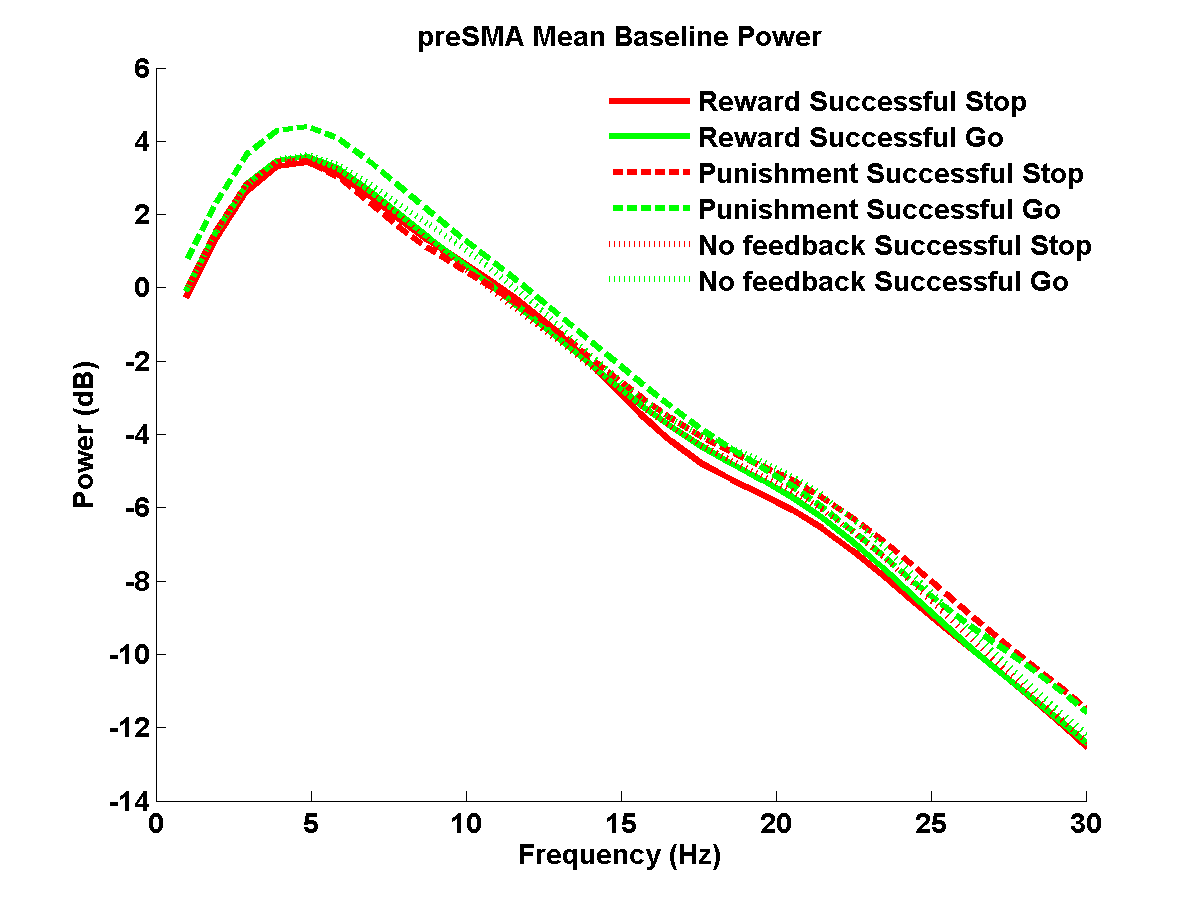


**Supplementary Figure 5.** Mean Baseline power of successful stop (SS), successful go (SG) under preSMA.


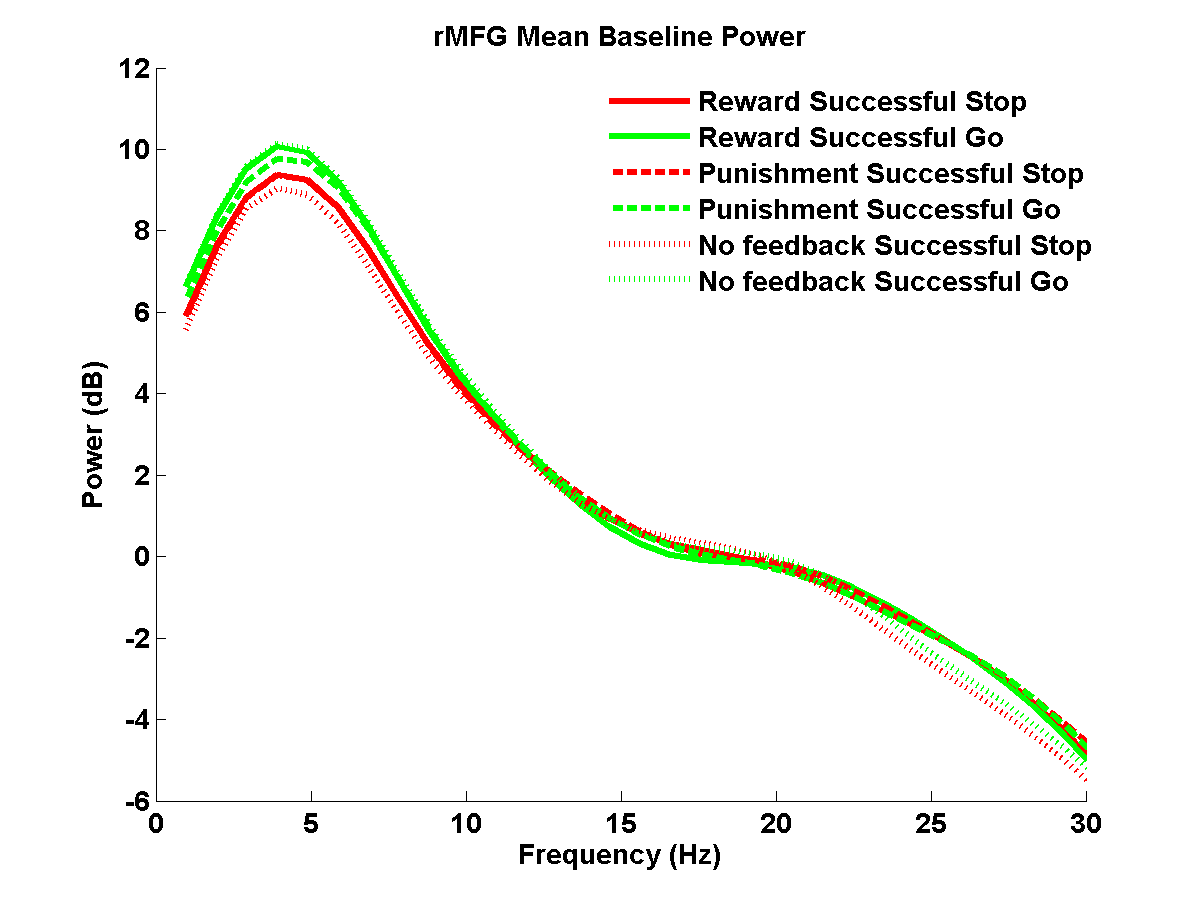


**Supplementary Figure 6.** Mean Baseline power of successful stop (SS), successful go (SG) under right MFG.

**
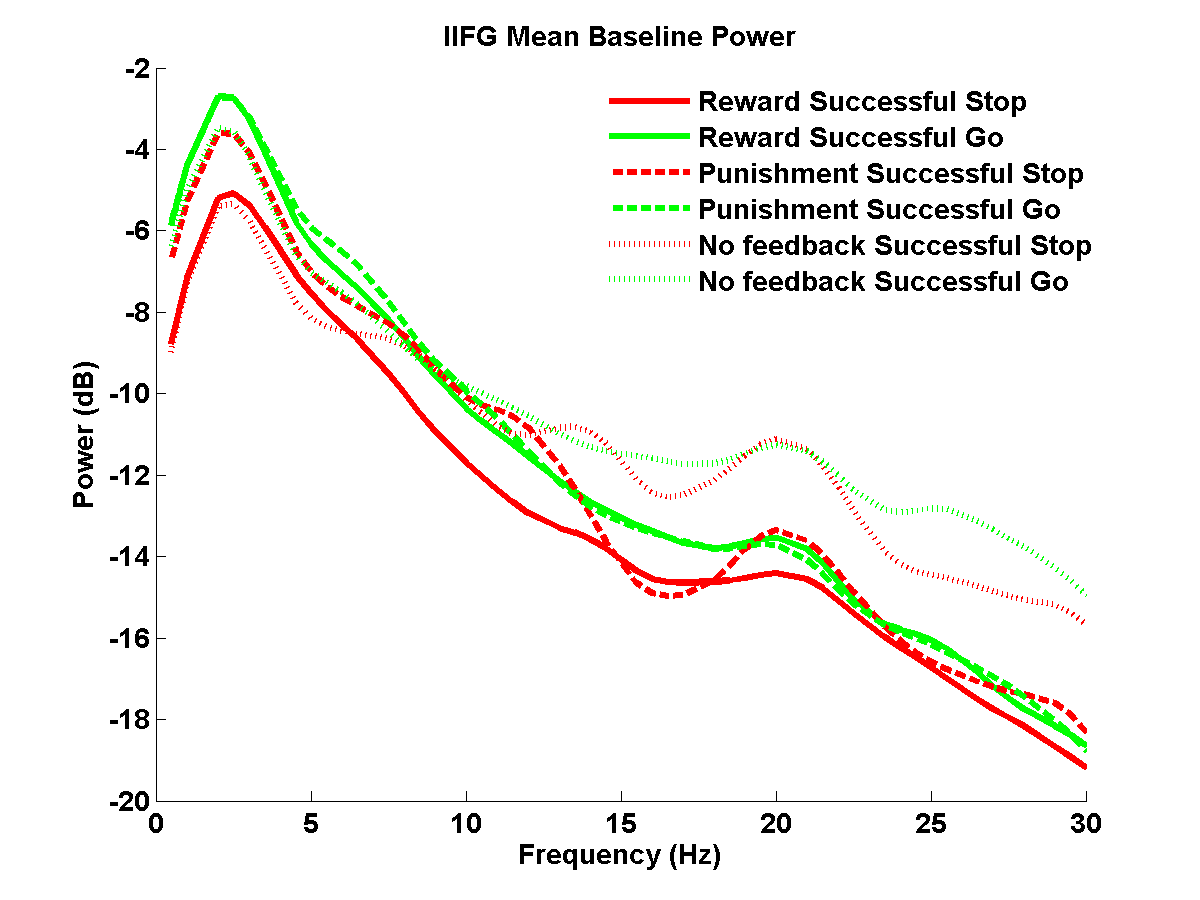
**

**Supplementary Figure 7.** Mean Baseline power of successful stop (SS), successful go (SG) under right lIFG.

**
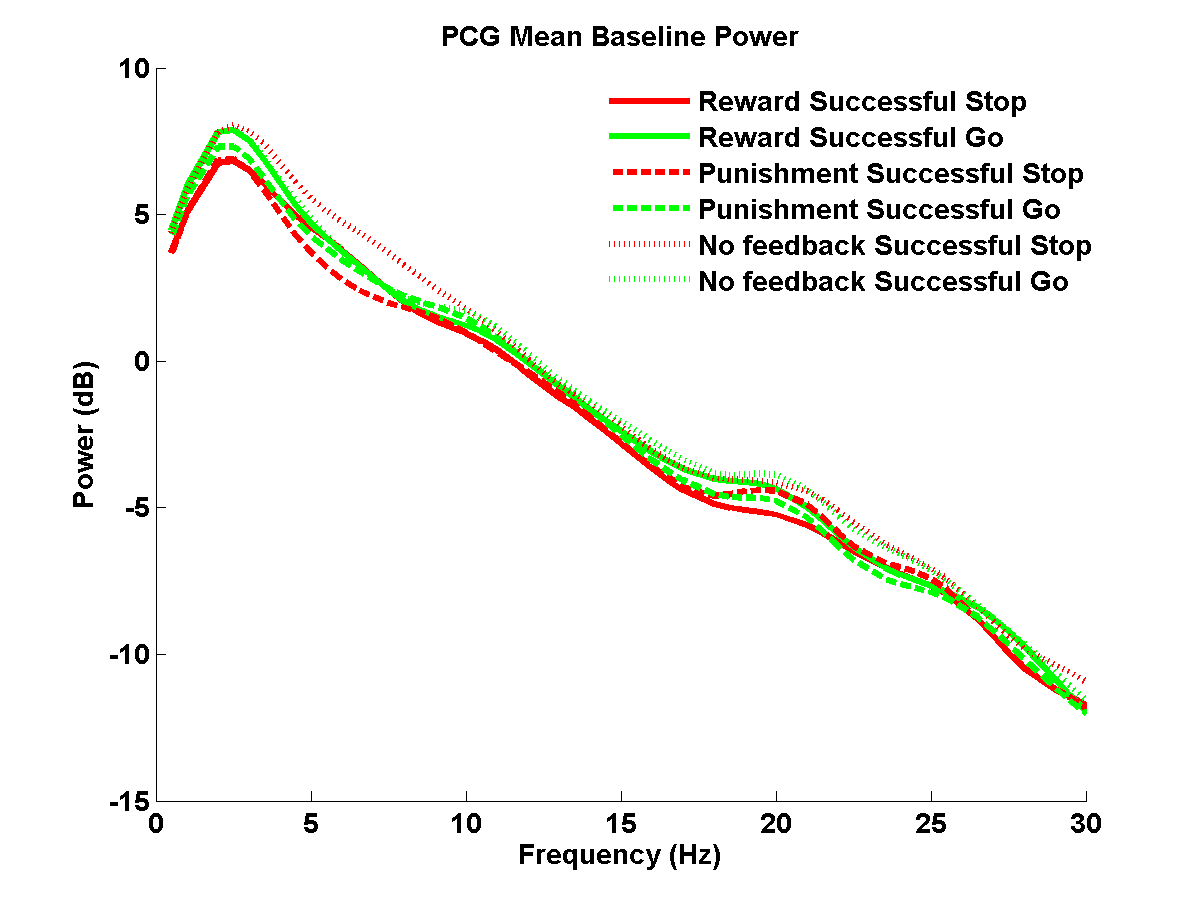
**

**Supplementary Figure 8.** Mean Baseline power of successful stop (SS), successful go (SG) under PCG.

**
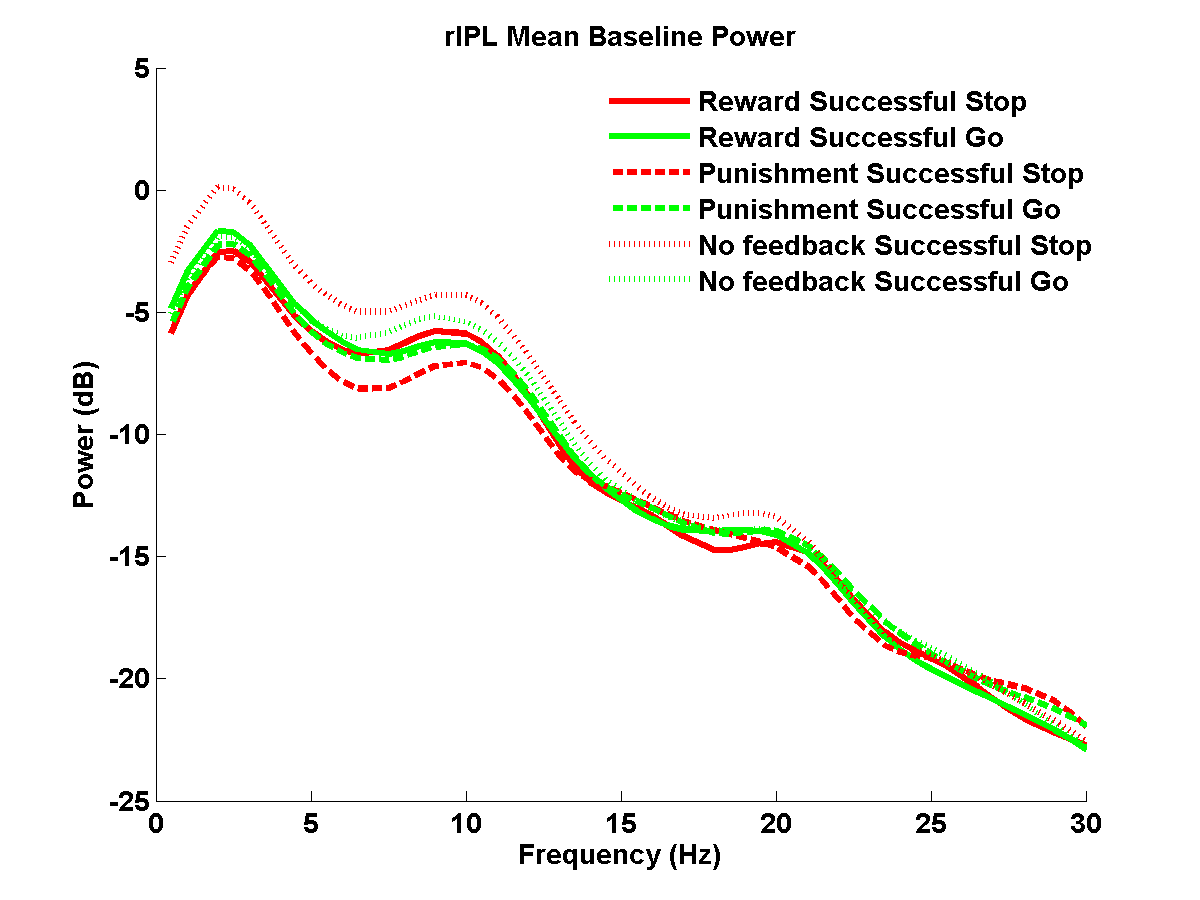
**

**Supplementary Figure 9.** Mean Baseline power of successful stop (SS), successful go (SG) under rIPL.

**
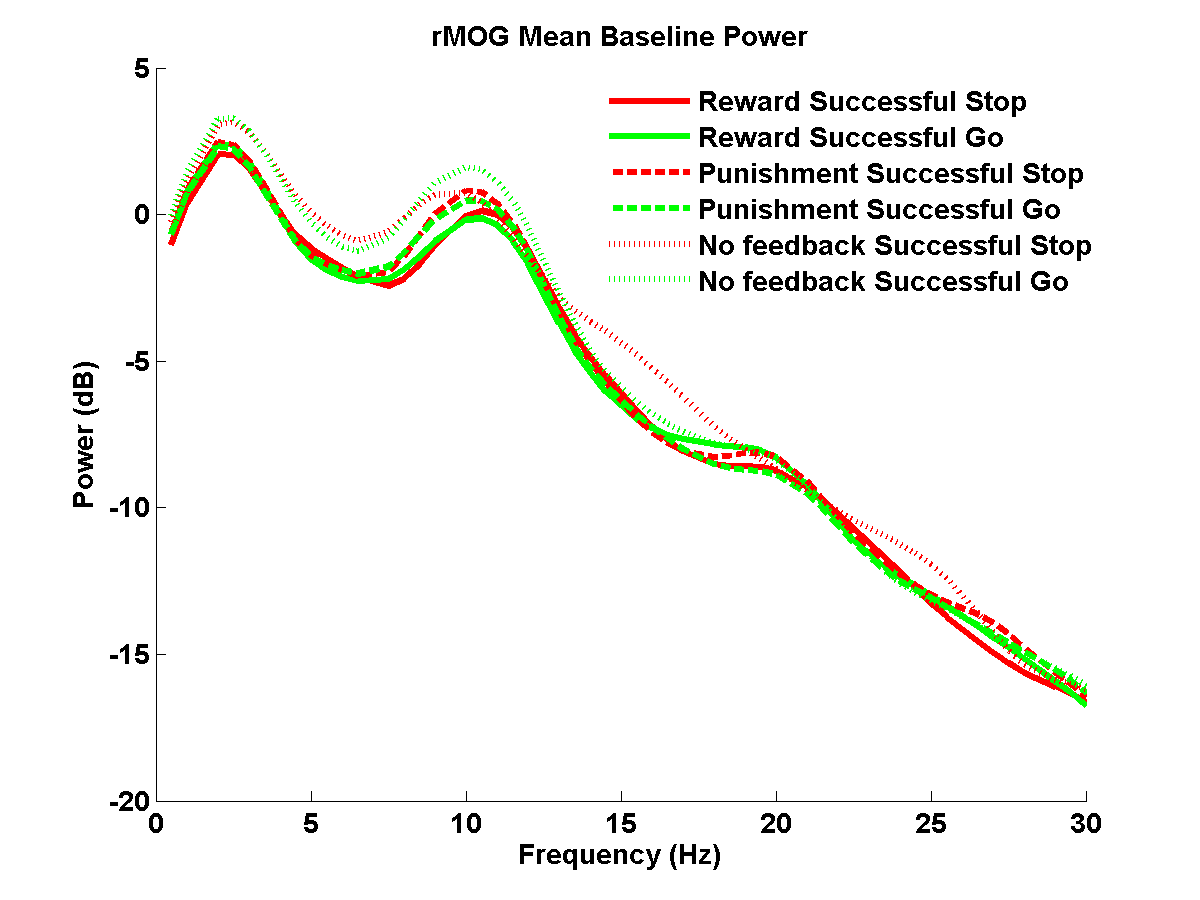
**

**Supplementary Figure 10.** Mean Baseline power of successful stop (SS), successful go (SG) under right MOG.

**
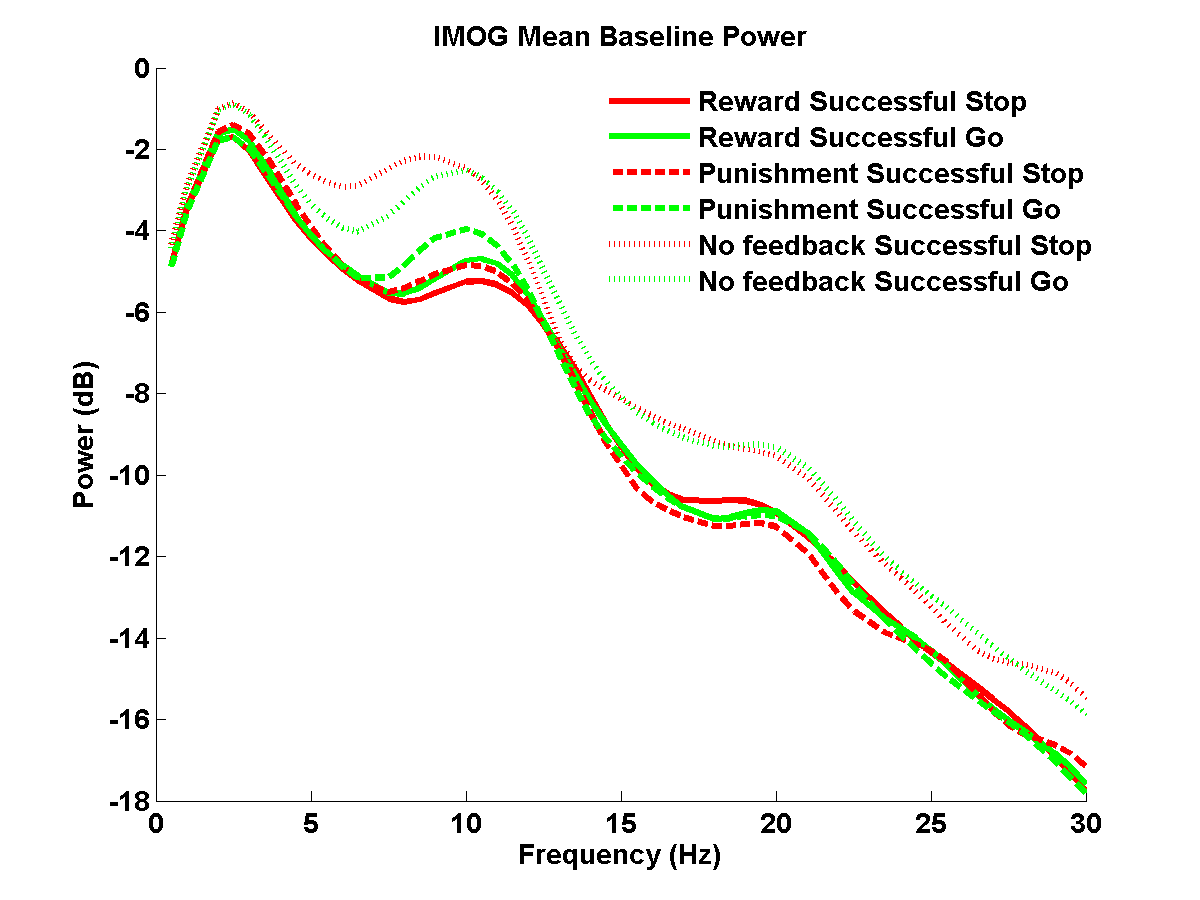
**

**Supplementary Figure 11.** Mean Baseline power of successful stop (SS), successful go (SG) under left MOG.
